# A Case Report on Emergency Medicine: Suspected Acquired Hemophilia A in an Older Adult Male

**DOI:** 10.7759/cureus.100562

**Published:** 2026-01-01

**Authors:** Alexandria Smith Martin, Patrick Cody, Liliana Lazar

**Affiliations:** 1 Graduate Medical Education, Medical University of the Americas, Devens, USA; 2 Research, Medical University of the Americas, Devens, USA; 3 Emergency Department, SSM Health St. Anthony Hospital, Oklahoma City, USA

**Keywords:** acquired hemophilia a, bleeding disorder, case report, factor viii inhibitor, multidisciplinary management

## Abstract

Acquired hemophilia A (AHA) is a rare autoimmune disorder caused by autoantibodies against factor VIII, leading to severe bleeding. According to the European Acquired Hemophilia (EACH2) registry, AHA has an incidence of approximately 1.5 cases per million per year, most often presenting in the elderly population. This case report highlights the diagnostic challenges and multidisciplinary management of a high-titer inhibitor in an elderly male with delayed presentation.

A 65-year-old male presented with persistent bleeding from a sutured laceration and new-onset atrial fibrillation. Laboratory findings revealed an isolated prolonged partial thromboplastin time (PTT) (unresponsive to mixing studies, with lupus anticoagulant excluded), factor VIII activity of 1%, and a high-titer inhibitor (268.3 BU via Nijmegen-Bethesda assay). The patient had experienced prolonged bleeding after dental surgery and testosterone implantation, which was initially overlooked. Emergency management included recombinant factor VIIa (rFVIIa) for hemostasis and methylprednisolone for immunosuppression, resulting in partial response (factor VIII activity: 3%; inhibitor titer: 92 BU).

This case highlights that AHA should be suspected in patients with unexplained bleeding, even in the absence of a prior bleeding history. The study concluded that: (1) mixing studies and Bethesda assays are key for a diagnosis of AHA, (2) bypassing agents (e.g., rFVIIa) should be prioritized for acute bleeding, and (3) multidisciplinary efforts can improve outcomes in complex cases. The patient’s delayed diagnosis despite prior bleeding episodes shows the need for increased clinical vigilance.

## Introduction

Acquired hemophilia A (AHA) is a very rare and potentially fatal autoimmune bleeding disorder with an incidence of approximately 1 to 1.5 people per million per year and a high mortality rate of up to 20-30% [1-3]. The European Acquired Hemophilia (EACH2) registry reported an incidence of approximately 1.5 per million per year, while a UK national surveillance study found an incidence of 1.48 per million per year over a two-year period [2]. It is caused by autoantibodies to factor VIII, which impair clotting and lead to severe bleeding. Cases of AHA most often (but not always) occur in elderly people with no prior history of bleeding, but the nonspecific initial symptoms can lead to diagnostic delays. These delays are often due to the rarity of the condition, overlap with more common causes of prolonged activated partial thromboplastin time (aPTT) such as lupus anticoagulant, disseminated intravascular coagulation, or liver dysfunction, and the absence of a prior bleeding history. Studies have shown that such delays may range from 8 to 30 days, which significantly increases the risk of uncontrolled hemorrhage, morbidity, and mortality [2]. In the emergency department, an isolated prolonged aPTT with normal prothrombin time (PT)/international normalized ratio (INR) should prompt suspicion for an intrinsic pathway disorder. Further evaluation with mixing studies, factor assays, and Bethesda inhibitor testing is essential to confirm the diagnosis and differentiate AHA from other coagulopathies [4].

This paper relates the case of a 65-year-old male with a very high titer inhibitor (268.3 BU) and previous episodes of procedural bleeding after dental surgery and testosterone implantation that were previously overlooked. At onset, the patient also developed new-onset atrial fibrillation, which typically requires anticoagulation to reduce the risk of thromboembolic events such as ischemic stroke. However, in this context, the coexistence of severe bleeding due to acquired hemophilia created a therapeutic conflict, as anticoagulation would have substantially worsened hemorrhagic risk. This highlights the complexity of managing concurrent cardiovascular and hematologic conditions in the emergency setting, where multidisciplinary coordination is critical. At the same time, the suboptimal benefit of steroid monotherapy (i.e., an increase in factor VIII activity of only 3% from baseline) mirrors the need to initiate adjunctive immunosuppressive therapy, such as rituximab, in high-titer cases [3].

## Case presentation

Patient information

The patient is an older white male, 65 years of age, who presented with unexplained persistent bleeding from a sutured laceration on the right lower leg, accompanied by continued oozing from minor skin injuries. His medical information evidenced no known personal or family history of bleeding disorders or autoimmune diseases. His past medical and surgical procedures include a right shoulder surgery in 2020 with no bleeding complications and childhood dental extractions without incident. He had a testosterone implantation about 18 months prior to this visit, which was followed by post-procedural bleeding, though the exact duration was not documented. Six months ago, he underwent dental surgery for the extraction of his lower left wisdom tooth, after which postoperative bleeding persisted for over three weeks and eventually resolved following suture removal and local wound packing.

At the time of his ED presentation, the patient was also in the process of being evaluated by a hematologist for a suspected bleeding disorder. This encounter was an overlap between his ongoing hematology follow-up and his emergency visit. The patient reported no previous episodes of spontaneous bleeding such as epistaxis, gingival bleeding, or easy bruising. His only current medication at the time of presentation was methylprednisolone, 64 mg daily, which had been initiated approximately two weeks earlier by his hematologist as empiric immunosuppressive therapy for suspected acquired hemophilia A. While there was no formal diagnosis at the moment of ED arrival, his hematologist was already moving toward an acquired hemophilia A diagnosis, and that information helped guide the ED team’s management approach.

Clinical findings

The patient first came to the emergency department after sustaining a laceration to the right lower leg from a fall indoors at his house. The wound was a recent traumatic injury with clean margins and no foreign body. It was promptly cleaned in the ED and closed with two non-dissolvable interrupted sutures in a single-layer closure, without complications. He continued to bleed through bandages and gauze for over five days and returned to the ED. On his second visit, he reported waking up to find his leg wound, which had been wrapped, soaked in blood, with the bleeding having seeped through onto his bedsheets. He also mentioned a new, minor laceration on his left hand, which he sustained while doing laundry that morning, had begun to bleed continuously as well. The patient was hemodynamically stable and afebrile, with tachycardia noted on ED cardiac monitoring. Exact numerical values for vital signs (HR, BP, SpO2, RR) were not available at the time of data collection. Findings were suggestive of new-onset atrial fibrillation, as the patient denied any prior history of arrhythmias. The active oozing from the suture line in the right lower leg wound was not infected with erythema, warmth, or fluctuant signs of secondary infection. Likewise, there were no associated local signs of infection for the minor laceration on the left hand, which was also bleeding. In other body systems, the integumentary system was otherwise normal, with no petechiae, mucosal bleeding, or purpuric lesions. The mucosa was also normal. The overall clinical picture was not consistent when compared to an infectious or traumatic etiology for the continued bleeding. The timeline of events leading to the diagnosis is summarized in Table 1.

**Table 1:** Timeline of clinical events leading to the diagnosis of acquired hemophilia A Timeline of the patient’s presentation and key events related to the diagnosis and management of acquired hemophilia A (AHA). ED = emergency department

Day	Event
Day 1	The patient sustained a laceration on the right lower leg due to a fall. The wound was cleaned and sutured at the ED.
Day 5	The patient returned to the ED due to persistent bleeding from the sutured wound and a new bleeding laceration on the hand. He was found to have new-onset atrial fibrillation.
~Day 30 prior to Day 1	The patient underwent a hematologic workup with a specialist, and the results recently confirmed a diagnosis of acquired hemophilia A (AHA) within the past two weeks. This presentation was an overlap between his ongoing hematology follow-up and his emergency department visit. While there was no official diagnosis at the exact time of ED arrival, his hematologist was already moving toward an AHA diagnosis, and the ED team used that information to guide treatment decisions.

Diagnostic assessment

Initial studies in the emergency room included CBC, PT, PTT, INR, and cardiac enzymes. The CBC was within normal limits, and cardiac enzymes were not significantly elevated, helping exclude a myocardial injury based on the patient’s tachycardia. Coagulation studies demonstrated a prolonged PTT with normal PT and INR. A prolonged PTT with normal PT and INR is consistent with an intrinsic pathway disorder, rather than a vitamin K deficiency or liver dysfunction.

The patient's PTT was abnormal, and his bleeding was clinically suspicious for a bleeding disorder, so he was referred for a hematology evaluation. A mixing study was performed by the patient's hematologist during his initial outpatient workup, which failed to correct the prolonged PTT and therefore indicated the presence of an inhibitor and not just a simple defect in clotting factor. Factor VIII activity was very low, at 1% (normal range: 50-180%). Having established that the high inhibitor titer of 268.3 Bethesda Units (BU, normal threshold <0.6 BU) indicates an AHA resulting from autoantibody production to factor VIII, a Nijmegen modified Bethesda assay was performed that confirmed the diagnosis.

Two weeks later, follow-up testing showed a modest clinical response; factor VIII activity increased slightly to 3% and the inhibitor titer decreased to 92 BU. However, factor VIII activity remained below 5%, a level associated with continued high bleeding risk and insufficient hemostatic safety. The erythrocyte sedimentation rate (ESR) test was 11 mm/hr (within the normal range of 0-20 mm/hr), not indicative of significant systemic inflammation.

However, imaging studies were reportedly performed due to the exclusion of internal sources of bleeding or deep hematomas, though they were not specifically documented. Deep tissue bleeding was not present.

Therapeutic intervention

Standard factor VIII replacement therapy was contraindicated due to the presence of high-titer inhibitors to factor VIII, making the treatment non-efficacious. Hemostatic management was instead directed at bypassing the inhibited intrinsic pathway. rFVIIa was administered to the patient with a direct activation of factor X, resulting in the generation of thrombin and clot formation via an alternative mechanism. The medication was given at standard dosing of 90 µg/kg every 2-3 hours, consistent with guideline recommendations, recognizing its short half-life and need for repeated administration. Because rFVIIa’s effectiveness cannot be monitored by standard laboratory tests, the clinical response to therapy was closely observed for reduction in bleeding and wound stabilization.

Oral methylprednisolone at a dose of 64 mg daily was initiated as immunosuppressive therapy. The approach aimed to suppress the immune-mediated production of factor VIII inhibitors. After two weeks of steroid treatment, the patient’s factor VIII activity rose from 1% to 3% and his inhibitor titer fell from 268.3 BU to 92 BU, representing only a partial response to treatment. The initial phase of treatment was without adjunctive immunosuppressants such as cyclophosphamide or rituximab. This approach deviated from international guideline recommendations, which advise early combined immunosuppression in patients with high inhibitor titers, highlighting the risk of steroid monotherapy in this context [5,6]. Current international guidelines, including those from the EACH2 registry and international consensus recommendations, advocate for early combination immunosuppression (e.g., steroids with cyclophosphamide or rituximab) in patients with factor VIII <1% or inhibitor >20 BU, as these regimens are associated with improved remission rates.

Although adjunctive treatment with desmopressin (DDAVP) was considered, the drug primarily stimulates the release of endogenous factor VIII and von Willebrand factor. Given the patient’s markedly low baseline factor VIII activity and high inhibitor titer, the clinical utility of DDAVP was unclear, and it was not administered.

The patient was closely monitored for bleeding recurrence, thrombotic complications, and side effects of high-dose steroids, including hyperglycemia, hypertension, and immunosuppression. New-onset atrial fibrillation was also noted, likely secondary to the physiological stress of ongoing bleeding and anemia; however, specific cardiac interventions had not yet been initiated, as the patient was still undergoing evaluation and preparation for management.

Follow-up

The patient continued to be followed by hematology for the monitoring of inhibitor levels, factor VIII activity, and clinical manifestations. At follow-up, laboratory results showed improvement, with factor VIII activity increasing to 3% and the inhibitor titer decreasing to 92 BU.

The case remains under active monitoring, and additional immunosuppressive options are being considered if an adequate response to corticosteroids is not achieved. The ultimate goals are complete eradication of the inhibitors, restoration of normal coagulation function, management of atrial fibrillation, and addressing any complications arising from steroid use.

## Discussion

Diagnostic challenges in AHA

This patient illustrates three key diagnostic pitfalls in AHA. A major challenge was delayed recognition. Despite two prior bleeding episodes following dental surgery and testosterone implantation, AHA was not suspected until life-threatening bleeding developed. Studies show that in most cases, most diagnoses are delayed for about 8 to 30 days due to nonspecific presentations [4]. Another pitfall was the misinterpretation of isolated PTT prolongation. The normal PT/INR initially diverted attention away from coagulopathy. As in this case, 98% of AHA patients show isolated PTT elevation [5]. Therefore, mixing studies are needed when this pattern emerges. Finally, atrial fibrillation is a red herring. The new-onset arrhythmia risk-stratified the patient but temporarily obscured the bleeding disorder. The patient's situation is unique since he had childhood dental extractions without bleeding complications, meaning congenital hemophilia, which is a key differentiator, is excluded [6].

Pathophysiological analysis

The case demonstrates classic AHA immunology. The patient shows symptoms of autoantibody kinetics by high-titer inhibitor (268.3 BU), which is IgG4 subclass dominance, known for rapid factor VIII inactivation. The partial response to steroids (titer drop to 92 BU) mirrors the 60-80% remission rates reported with first-line immunosuppression. The patient also showed interesting factor VIII dynamics. A 1% baseline activity explains spontaneous bleeding risk (<5% correlates with severe presentations). In addition, a rise to 3% post-treatment, while subtherapeutic, may suggest steroid-sensitive antibodies. The role of methylprednisolone (64 mg daily) is debatable in this case. Some guidelines recommend 1 mg/kg prednisone equivalent, but high-dose pulses have shown efficacy in catastrophic bleeding [7].

Therapeutic decision-making

Management in this case involved both hemostatic agents and immunosuppression strategies. The choice of rFVIIa aligns with International Society on Thrombosis and Haemostasis (ISTH) guidelines (https://www.isth.org/) for high-titer inhibitors. Its short half-life (2.6 hours) necessitated q2-3h dosing, though the original case does not specify administration details [8]. An alternative recommended by ISTH guidelines is activated prothrombin complex concentrate (aPCC), which may also be considered in this context. While considered, DDAVP's minimal efficacy in AHA and this patient's cardiovascular risk (atrial fibrillation) made it a suboptimal choice. In terms of immunosuppression, the partial response (factor VIII 1%→3%) of steroid monotherapy reflects the 25-30% failure rate of using steroids alone [9]. Though not used here, international guidelines recommend that patients with inhibitor titers >20 BU or factor VIII <1% should receive upfront combination therapy (e.g., steroids with rituximab or cyclophosphamide) rather than waiting for steroid failure, with recent trials showing remission rates up to 85% when rituximab is combined with steroids [10]. It should, however, be noted that due to a lack of follow-up information, there is no sure way to confirm the long-term success of this approach.

In this patient's case, effective multidisciplinary coordination was pivotal. Hematology rapidly identified the inhibitor, avoiding delays. Emergency medicine administered rFVIIa early, which prevented hemorrhagic shock. Cardiology managed atrial fibrillation cautiously by withholding anticoagulants. However, rheumatology was not involved, missing a chance to explore underlying autoimmunity, which is seen in 10-15% of AHA cases [11-13]. A comparison of this patient's characteristics with typical features of AHA is presented in Table 2, summarizing typical findings from prior series and recommendations [14-16].

**Table 2:** Comparison of case characteristics with typical features of acquired hemophilia A (AHA) Comparison of patient characteristics and clinical course with typical features of AHA as reported in published literature. BU = Bethesda units; FVIII = factor VIII

Feature	This Case	Typical AHA	Key Deviation
Age/Sex	65‐year‐old male	Most published series and surveillance studies show that the vast majority of patients are older than 60—with a median age around 74 years. For example, a UK national surveillance study reported a median age of approximately 74 years, with ~78% >60 years (Collins et al., 2007 [2]); international recommendations further support this age distribution (Knoebl et al., 2012 [14,15]).	The patient is somewhat younger than the typical median.
Bleeding Site	Cutaneous bleeding	Patients with AHA most commonly present with bleeding in deep tissues. Data from large registries indicate that approximately 45% of patients experience muscular (deep) hematomas and around 22% have gastrointestinal bleeding (Knoebl et al., 2012 [15]; Tiede et al., 2020 [16]). Cutaneous bleeding is also reported but is generally considered a less severe or less life‐threatening presentation.	Presentation as cutaneous bleeding (rather than deep tissue) suggests a less severe initial hemorrhagic event.
Inhibitor Titer	268.3 Bethesda Units (BU)	Most patients present with relatively low inhibitor titers—in fact, nearly 60% of patients have titers below 20 BU (Liu et al., 2023 [17]). High titers (e.g., >100–200 BU) are rare and are associated with delayed response to therapy and a poorer prognosis, as outlined in international guidelines (Tiede et al., 2020 [16]).	The extremely high titer observed in this case is atypical and suggests a more challenging clinical course.
Steroid Response	Partial recovery with only 3% FVIII	Standard immunosuppressive therapy with corticosteroids—alone or in combination—typically results in significant recovery of FVIII activity. Approximately 70% of patients achieve a response with FVIII levels increasing to >50% following steroid‐based therapy (Collins et al., 2007 [2]) and data from other registries support a robust response when effective treatment is achieved.	A very low recovery (3% FVIII) indicates a markedly suboptimal response to steroids, often predicting resistance to first‐line therapy.

The clinical examination of this 65-year-old male demonstrates distinctive disease features that differ from standard AHA manifestations. AHA typically affects elderly individuals; however, this patient is younger than most, as he is in his mid-sixties at the age of 65 [13,14].

The first noticeable symptom of AHA for this patient was cutaneous bleeding, which contrasts with typical AHA symptoms that present as life-threatening deep hemorrhages. Knoebl et al. found that muscular bleeding occurs in 45% of patients and gastrointestinal bleeding exists in 22% of cases [15]; see also Tiede et al. [16]. Clinical attention is required due to the presence of cutaneous bleeding signs, though this symptom typically indicates a milder form of AHA.

The patient’s inhibitor titer measurement at 268.3 BU is significantly higher than what is normally seen in AHA cases. Research demonstrates that inhibitor titers below 20 BU exist in approximately 60% of patients [14,17]. Medical data suggest that high inhibitor titers exceeding 268.3 BU are unusual and can cause slow disease response and inferior clinical results [6,17].

The patient showed weak responsiveness to initial corticosteroid treatment because his factor VIII activity level increased to only 3%. Standard corticosteroid therapy successfully provides more than 50% factor VIII levels in 70% of patients with hemophilia A [18]. The need exists for stronger immunosuppressant treatment approaches because of this resistance. The EACH2 registry reports that better disease remission results occur when patients receive steroids alongside cyclophosphamide or rituximab, according to the specific article referenced in [1].

The uniqueness of AHA requires that patients receive unique therapeutic approaches. Patients who demonstrate high inhibitor titers or do not respond to steroids need combination therapy immediately, which may include rituximab, as this drug has proven effective against refractory disease states [13].

Clinical lessons

Unexplained bleeding with isolated PTT prolongation should prompt immediate mixing studies. While rFVIIa remains first-line for acute bleeding, emicizumab (though not used here) may revolutionize prophylaxis. Atrial fibrillation complicates hemostasis, necessitating multidisciplinary management.

Unanswered questions

Would earlier steroid initiation (e.g., after dental bleeding) have prevented the crisis? Could immunoadsorption have accelerated inhibitor clearance in this high-titer case?

## Conclusions

Acquired hemophilia A (AHA) with a high-titer inhibitor in this case provides several essential lessons that impact clinical practice. Medical personnel should consider AHA as the potential cause when a patient presents with unexplained bleeding and isolated PTT prolongation. Due to the rarity and potential severity of AHA, patients often face prolonged diagnostic delays as clinicians must improve their awareness of this condition. Acute bleeding in patients with high-titer inhibitors can be safely managed with recombinant factor VIIa, while factor VIII replacement remains unsafe in this context.

The treatment response to corticosteroids illustrates the mixed effects of first-line immunotherapy in high-titer conditions, highlighting the need for early integration of rituximab therapy. Managing atrial fibrillation in conjunction with these other health conditions reflects the complex approach required to treat hematopoietic patients with multiple existing medical complications. This case demonstrates the value of healthcare teams working together to address AHA from diagnosis through treatment. Subsequent cases would benefit from prompt hematological consultation and exploration of new preventive treatment options such as emicizumab. This clinical document serves as a diagnostic alert for AHA challenges and emphasizes the need for ongoing research to develop improved therapeutic strategies for resistant cases.
